# Is it Really Nature That Restores People? A Comparison With Historical Sites With High Restorative Potential

**DOI:** 10.3389/fpsyg.2018.02742

**Published:** 2019-01-28

**Authors:** Massimiliano Scopelliti, Giuseppe Carrus, Marino Bonaiuto

**Affiliations:** ^1^Department of Human Studies, LUMSA University, Rome, Italy; ^2^Centre for Interuniversity Research on Environmental Psychology (CIRPA), Rome, Italy; ^3^Experimental Psychology Laboratory, Department of Education, Roma Tre University, Rome, Italy; ^4^Department of Psychology of Developmental and Socialization Processes, Sapienza University of Rome, Rome, Italy

**Keywords:** nature, restorative environments, built/historical environments, on-site experience, well-being

## Abstract

Research on restorative environments has showed the healthy outcomes of nature experience, though often by comparing attractive natural to unattractive built environments. Some studies indeed showed the restorative value of artistic/historical settings. In a quasi-experimental study involving 125 participants in Rome, Italy, a natural and a built/historical environment, both scoring high in restorative properties, were evaluated in a natural, built/historical, or neutral setting. In accordance with the Biophilia hypothesis and the Attention Restoration Theory (ART), we hypothesized: a higher restorative potential of nature also when compared to built/historical environments; a moderation effect of on-site experience on perceived restorative potential (PRP) of both environmental typologies; higher levels of restorative properties of the environment for on-site vs. not on-site respondents; and a mediation effect of the restorative properties of the environment in the relationship between time spent on-site and PRP. Results supported the hypotheses. In addition, different psychological processes leading to restoration emerged for the natural and the built/historical environment. Theoretical implications for ART and practical applications for an integrative urban design with natural and historical elements are discussed.

## Introduction

Contact with nature has been widely recognized to promote health and well-being (Bratman et al., 2012; Hartig et al., 2014). The restorative potential of nature has been often used as a theoretical framework explaining these benefits (Kaplan, 1995). Psychological restoration refers to the capacity for natural environments to replenish cognitive resources depleted by everyday activities and to reduce stress levels, according to the Attention Restoration Theory (ART, Kaplan and Kaplan, 1989) and the Stress Reduction Theory (Ulrich, 1983), respectively. The two theories share an evolutionary approach rooted in the Biophilia hypothesis (Kellert and Wilson, 1993), which postulated that human beings have developed an innate tendency to positively respond to natural environments for adaptation reasons, because nature is the environment in which they evolved. Both ART and SRT stressed indeed the role of attention and low levels of stress for human survival and adaptation. Empirical evidence supporting the cognitive and affective benefits of contact with nature has been widely provided (Ulrich et al., 1991; Hartig et al., 2003; Herzog et al., 2003; Laumann et al., 2003; Staats et al., 2003; van den Berg et al., 2003; Kaplan and Berman, 2010).

In their seminal work on restorative environments, Kaplan and Kaplan (1989) have pointed out the importance of four *restorative properties* of nature in promoting positive outcomes, namely being-away, referring to a change of scenery and/or experience from daily routines, promoting a conceptual distance from the ordinary; fascination, intended as the capability of nature to involuntarily elicit the individual’s attention, without mental effort and thus the depletion of cognitive resources; extent, implying the properties of coherence among the environmental elements and scope in environments, which should be perceived as extended enough to engage the mind; and compatibility, which has to do with the perceived congruence between the characteristics of the environment and people’s needs, intentions and inclinations.

As such, the restorative properties may characterize any typology of environments – not only nature – ranging from completely natural to completely built. Kaplan and Kaplan (1989) clearly stated that also built environments showing high levels of these properties can have potential for restoration. Yet, because of their evolutionary approach, they also claimed that, beyond the levels of the restorative properties, it is the natural character of the environment itself to promote higher levels of psychological restoration. Surprisingly, and in spite of the huge literature accumulated on restorative environments since then, this hypothesis has never been tested. In other words, if environments promote restoration through their restorative properties, it should be expected that natural and built environments with comparable levels of restorative properties would bring equal benefits to their visitors. Conversely, if evolutionary theories hold, it should be expected a higher level of restoration for natural vs. built environments also in the condition of similar levels of restorative properties. As pointed out by Scopelliti and Giuliani (2004), built environments within this framework have often been studied in the urban context and opposed to natural environments in an unfair dichotomy. Several studies, indeed, have investigated the restorative potential of nature through a comparison between pleasant natural environments and unpleasant built environments (Purcell et al., 2001; Berto, 2005). In this regard, some authors have shown the role of perceived attractiveness, or similar concepts, in the restoration process (Dijkstra et al., 2008; Nasar and Terzano, 2010; Twedt et al., 2016). On the other hand, the study of the restorative potential of built environments is still scant, and referred to examples with historical and/or artistic value, such as museums, monasteries, renewed and attractive neighborhoods, and plazas (Kaplan et al., 1993; Scopelliti and Giuliani, 2004, 2005; Ouellette et al., 2005; Karmanov and Hamel, 2008; Abdulkarim and Nasar, 2014).

In addition, the role of place experience in promoting human well-being and health has been extensively recognized for natural environments in a variety of settings, populations, and individual conditions. The seminal study by Ulrich (1984) has showed the positive effect of visual contact with nature on the speed of recovering from surgery, and Hartig (2006) provided a thorough analysis of the potential influence of healing gardens in health care settings. In addition, de Vries et al. (2003) found that living in a green environment is positively associated to different health indicators, and this relationship is even stronger for specific groups of residents, namely housewives and elderly people. Frumkin (2001) discussed the potential benefits for human health deriving from the relationship with natural environments, and suggested the importance to join research findings to interventions in everyday settings. Milligan and Bingley (2007) debated the role of childhood woodland experience in strengthening personal resources against young adulthood difficulties, thus promoting mental health. Korpela and Ylén (2007) clearly pointed out that people choose natural place experiences for the self-regulation of mood, and their selection is influenced by - and influences - perceived health. An impressive epidemiological study by Mitchell and Popham (2008) on the population of England has showed that contact with nature decreased income-related health inequalities, promoting lower levels of circulatory disease and overall mortality for low-income people. On the whole, a noticeable bulk of studies addressed the issue of the beneficial effects of the relationship with nature, which seems to be more important in urban settings, where stressful situations and psychological demands for residents are more likely to come in conflict with the “pursuit “of urban sustainability (van den Berg et al., 2007).

Taking as a starting point the psychological literature on the experience of nature (e.g., Kaplan and Talbot, 1983; Talbot and Kaplan, 1986; Hull and Stewart, 1992; Hull and Michael, 1995; Stewart, 1998; Hammitt, 2000; Borrie and Roggenbuck, 2001), a central concern in the study of restorative environments was to develop rating scale measures of Perceived Restorativeness (PR), namely the level of restorative components attributed to environments, through the operalisation of the constructs of being-away, fascination, extent, and compatibility. With reference to this issue, the Perceived Restorativeness Scale (PRS; Hartig et al., 1996, 1997), the Restorative Component Scale – as named by Herzog et al. (2003) – (RCS; Laumann et al., 2001), and the Restoration Scale (RS; Han, 2003) have been developed. PR has been extensively considered in studies on the evaluation of environments, showing a positive relation with other affective and behavioral reactions: in Hartig et al. (1991) study, convergent validity emerged from a multi-method approach in which self-reports of affective states – the Zuckerman Inventory of Personal Reactions (ZIPERS, Zuckerman, 1977) and the Ontario Health Survey (OHS, Campbell et al., 1976) – a cognitive performance task and physiological measures of stress were employed with an earlier version of PRS. In the PRS validation studies by Hartig et al. (1996, 1997), the PRS subscales have showed a positive correlation with positive affect and a negative correlation with negative affect measured through ZIPERS. Hartig et al. (1998) have found that differences in PR of near-home environments positively covary with self-reported measures of stress. Laumann et al. (2001) have outlined the relationship between PR and relaxation. Herzog et al. (2003) have found that the four restorative components predicted a self-report measure of perceived restorative potential (PRP) of environments, namely the perceived restoration of the ability to work effectively. Korpela et al. (2001) have shown that experiences in favorite places, scoring high on PRS, are used for an emotional regulation implying relaxation, enjoyment and avoidance of anger, sadness, and nervousness. Finally, studies on the rapid affective evaluation of environmental scenes have outlined a positive relationship between PR and positive emotions (Korpela et al., 2002a; Hietanen and Korpela, 2004; Hietanen et al., 2007). These results suggested how specific characteristics of environments are capable to involuntarily elicit those automatic mental processes whose role in everyday experience has been largely discussed by social and cognitive psychology in general (e.g., Bargh and Chartrand, 1999).

Most of the studies on restorative environments have emphasized the beneficial outcomes of person-environment transactions through a methodology that stressed the role of visual perception in the restoration process. Slides and photos, and sometimes videos have been the typical stimuli employed in experimental designs (e.g., Ulrich et al., 1991; Purcell et al., 2001; Herzog et al., 2003; Laumann et al., 2003; Staats et al., 2003; van den Berg et al., 2003; Staats and Hartig, 2004; Berto, 2005). Exceptions to this approach are sometimes available in the literature, both implying reconstruction of restorative experiences (Scopelliti and Giuliani, 2004, 2005) and actual experiences in the environment (Hartig et al., 1991; Bowler et al., 1999; Hartig et al., 2003; Morita et al., 2007; Barton et al., 2009; Berman et al., 2012; Marselle et al., 2014). Even though the representational validity of slides and videos for real environments has been repeatedly confirmed in the literature on environmental evaluation and landscape assessment (Zube, 1974; Shuttleworth, 1980; Kellomaki and Savolainen, 1984; Stewart et al., 1984; Stamps, 1990; Hetherington et al., 1993; Heft and Nasar, 2000), some authors have pointed out interesting theoretical issues referring to the typologies of environments which may be adequately represented through these presentation media. As Vining and Orland (1989, p. 281) stated, some “environments may not be successfully represented because of their symbolic or non-visual values,” so that “it is important to understand the cognitive and/or affective processes which lead to judgements.”

In this regard, a major claim in the research on restorative experience is that the restoration process may continue and get deeper through several stages, ranging from clearing one’s mind to renewing directed attention mechanism, to possibility for reflection on personal issues (Kaplan and Kaplan, 1989). In other words, it involves a dynamic of person-environment transaction in which the temporal dimension plays a fundamental role. Far from being only a matter of perception, restoration seems to be the consequence of a global place experience, where overall person-environment transactions are of importance (Scopelliti and Giuliani, 2004). That is, several aspects of actual person-environment transactions may have a moderation effect on positive outcomes. An empirical evidence of the differences in place evaluation when people are asked to “look at” the place or thinking about the molar place experience is provided by Scott and Canter (1997). In the same direction, Hull and Stewart (1992) have underlined the role played by mood, personal meanings and activities performed in the environment, when evaluations are of interest. With reference to restorative environments, Herzog et al. (2002) have showed the importance of a number of contextual factors in the evaluation and selection of settings for restoration; and both Scopelliti and Giuliani (2004, 2005) and Staats and Hartig (2004) have emphasized the effect of the social environment in restoration outcomes. In addition, recent studies have suggested that a relevant mechanism promoting positive outcomes can be the perception of the restorative properties of the environment during on-site experiences in urban parks (Scopelliti et al., 2016), botanical gardens (Carrus et al., 2017) and educational settings (Amicone et al., 2018). These results deserve further exploration in terms of psychological processes leading to restoration as the on-site experience goes on.

The analysis of personal experiences has addressed interesting research avenues for restorativeness studies: among the others, the relationships between restorative experiences, favorite places and the definition of personal identity (Korpela, 1992; Korpela and Hartig, 1996; Korpela et al., 2001; Korpela et al., 2002b); and the role of ecological activities in the restoration process and their influence on pro-environmental behavior (Bowler et al., 1999). All those issues considered, the present study has been developed to address several research questions.

The first aim was to further test the basic assumption of ART, SRT, and the overall Biophilia hypothesis about the intrinsic restorative potential of nature, particularly when comparing it with built/historical environments having high levels of restorative properties. It is hypothesized that (H1) high-quality natural environments would be perceived with a greater PRP also when compared to high-quality built environments (i.e., historical ones); these should, however, be capable to promote a moderate-to-high level of PRP.

The second aim was to better understand the added value of actual on-site experiences in restorative environments, as compared to mere perceptual experiences often investigated in experimental studies. On-site experiences take into account the overall person-environment transactions, also including social activities. It is hypothesized that (H2) on-site experiences in restorative environments should moderate PRP in different environmental scenes. In particular, when judging PRP in natural vs. built/historical environments, the difference in favor of natural scenes should be at the highest for people in a natural environmental experience. Conversely, the difference in favor of natural scenes should be at the lowest for people in a built/historical environmental experience. Finally, the difference in favor of natural scenes should be at an intermediate level for people in a neutral environmental experience.

The third aim was to gain a better understanding of the processes promoting restoration in real environmental experiences, with particular attention to the role of time spent in restorative environments. It is hypothesized that (H3) the added value of on-site experience in terms of PRP, in both natural and built/historical environments, is promoted by an increase in the perception of the restorative properties of the environment; and (H4) the increase of time spent on-site would promote higher level of PRP through an increase in the perception of the restorative properties of the environment.

A two-phase approach was followed. The first study was aimed at identifying natural and built/historical environments having a comparable level of PR. The second study was aimed at testing the above hypotheses.

## Materials and Methods

### Study 1

Study 1 was run in order to identify both natural and built environments with high restorative potential. The city of Rome was selected as the context for the study, because of its wide presence of both natural and built/historical places. In particular, 10 natural (Laghetto dell’Eur, Parco del Gianicolo, Parco dell’Appia Antica, Villa Ada, Villa Borghese, Villa Celimontana, Villa D’Este, Villa Lazzaroni, Villa Paganini, Villa Pamphili) and 10 built/historical places (Campo dei Fiori, Castel Sant’Angelo, Fontana di Trevi, Piazza della Rotonda al Pantheon, Piazza del Popolo, Piazza di Spagna, Piazza Navona, Piazza San Pietro, Piazza S. Maria in Cosmedin, Piazza Santa Maria in Trastevere) were selected. The presence of some extent of the place and fascinating elements (natural vs. artistic/historical) were relevant criteria for selection. Color photographs of the different environments were taken between 11 am and 2 pm in sunny days in spring, and showed both natural and built environments in similar light and crowding conditions.

#### Participants and Procedure

Sixty-two university students were contacted at the Department of Psychology of Developmental and Socialization Processes at Sapienza University of Rome, and asked to take part in the study. Forty students (27 females 17–63 years old), agreed to participate and were randomly assigned to five different groups. Each group was asked to rate the restorative potential of two natural and two built environments on a short Italian version of the PRS (Pasini et al., 2009), consisting of eight 5-step Likert-type items referring to being-away, fascination, extent, and compatibility. One more item was used to measure preference. The pictures of the settings (12 cm × 18 cm) were presented on a 15-inch monitor at the laboratory of the Department of Psychology of Developmental and Socialization Processes. Participants took about 5 min to fill in the questionnaire.

#### Results

Overall levels of PR of the twenty settings were calculated by computing a mean score of the restorative properties (see Table 1). On the whole, natural and built environments did not show a significant difference [*F*_(1,39)_ = 1.74, n.s.] in PR and in preference either [*F*_(1,39)_ = 1.56, n.s.].

**Table 1:** Rank order and perceived restorativeness scores of selected natural and built environments (Study 1).

Rank order	Natural environments	Mean	Rank order	Urban environments	Mean
2	Villa Celimontana	3.73	1	Piazza Navona	3.84
3	Villa Pamphili	3.58	6	Fontana di Trevi	3.32
4	Villa D’Este	3.50	7	Castel Sant’Angelo	3.29
5	Parco Appia Antica	3.44	12	Piazza di Spagna	3.01
8	Villa Borghese	3.24	13	Piazza S.Maria in Cosmedin	3.01
9	Laghetto dell’Eur	3.23	14	Campo dei Fiori	2.94
10	Villa Paganini	3.17	15	Piazza del Pantheon	2.84
11	Villa Ada	3.06	16	Piazza S.Maria in Trastevere	2.82
18	Villa Lazzaroni	2.72	17	Piazza del Popolo	2.79
20	Parco del Gianicolo	2.66	19	Piazza San Pietro	2.71
	TOTAL	3.23		TOTAL	3.06

**FIGURE 1:**
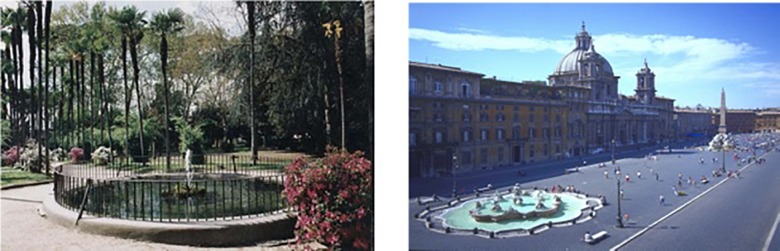
Pictures of Villa Celimontana (left) and Piazza Navona (right) used as stimuli.

In particular, no difference emerged between the most restorative natural and built environment (Villa Celimontana and Piazza Navona, respectively) in terms of PR [*t*_(14)_ = 1.16, n.s.]. Villa Celimontana is an urban park of about 110,000 square meters, located in the center of Rome. It is not as wide as others parks in Rome, but it is well equipped with pathways along rows of monumental trees, well-kept grass lawn, shrubs, and water. Piazza Navona is an historical square in Rome of about 11000 square meters, with a variety of artistic and historical elements, including Baroque churches, statues and fountains, and an obelisk. The pictures representing these two places were selected and color printed in 12 cm × 18 cm format (Figure 1) for the aims of Study 2, as they emerged to have similar restorative potential.

### Study 2

A quasi-experimental research design was set up. The main design variables were:

(1) The typology of scene represented in the pictures used as stimuli (Environment: Natural – Villa Celimontana vs. Built/historical – Piazza Navona), considered as a within-subjects factor.
(2) The typology of place where the participants evaluated the pictures (Condition: Natural vs. Built/historical vs. Neutral), considered as a between-subjects factor. The two main locations were the same places represented in the pictures used as stimuli, namely Villa Celimontana and Piazza Navona. A third neutral location was considered as a control condition, namely the laboratory of the Department of Psychology of Developmental and Socialization Processes at Sapienza University of Rome.

The dependent variables were the measures of the restorative properties of the two settings and a general measure of PRP.

#### Participants and Procedure

Participants were 125 subjects, either living in Rome or coming from other parts of Italy, aged 16–72 years, and well balanced with respect to gender (females: *N* = 62). Subjects were opportunistically selected for each experimental condition. For the “Neutral” condition, 74 students were contacted at the Department of Psychology of Developmental and Socialization Processes, Sapienza University of Rome but 24 did not agree to participate. We recruited 50 participants, aged 19–32 (mean = 22.10, *sd* = 3.48). In on-site conditions, people were visiting the environment, and spending time walking, relaxing, and socializing. For the natural condition (Villa Celimontana), 59 individuals were contacted on-site, but 22 did not agree to participate. We recruited 37 participants, aged 16–44 (mean = 24.92, *sd* = 5.55). For the built/historical environmental condition (Piazza Navona), 61 individuals were contacted on-site, but 23 did not agree to participate. We recruited 38 participants, aged 16–72 (mean = 30.89, *sd* = 14.00).

In each experimental condition, subjects were shown the pictures of the two environments on 12 cm × 18 cm photographs. The order of presentation of the pictures was randomized. Participants were first asked to identify the two places, and no difficulty in this task emerged. They were then asked to evaluate each environment in a quiet area of the setting through the Italian version of the PRS. Each picture was shown for 3 min and then kept in front of the respondent throughout the duration of the evaluation. Participants were also asked to judge the level of annoyance in the environment when performing the task. No expression of annoyance emerged. In both on-site situations, the study was carried out when light and crowding conditions were similar to those shown in the pictures. Given the selection method adopted, some possible relevant differences in the psychological characteristics of participants across experimental conditions (i.e., personality traits, individual differences, and environmental attitudes) were finally checked for, in order to rule out the possibility of a self-selection of subjects. Details on the tools we used are given in the following section. Data were gathered in sunny days in Spring 2015.

#### Measures

A self-report paper-and-pencil questionnaire composed by different tools was used for this study. The questionnaire was arranged as follows.

Section 1: Italian version of the PRS, measuring PR through 26 5-step Likert scale items referring to the constructs of being-away, fascination, extent, and compatibility; three more items measuring preference and familiarity were also included in the tool (Pasini et al., 2009). A single item measuring PRP, adapted from Herzog et al. (2003); (“Recall one of those times when you worked hard on a project that required intense and prolonged effort. How good would be the experience in this place to restore your ability to work effectively?”) was finally presented. All the above items were proposed for the evaluation of both Villa Celimontana and Piazza Navona. One item was used to measure perceived crowding of the environment only for on-site respondents in the natural and in the built/historical places.

Section 2: Italian version of the Big Five Questionnaire (BFQ, Caprara et al., 1993). In particular, a shorter version of the sub-scales referring to “Openness to Culture” and “Openness to Experience” (12 5-step Likert scale items) was used.

Section 3: Italian version of the Sensation Seeking Scale (Galeazzi et al., 2003). In particular, the sub-scale referring to “Experience Seeking” (10 dichotomous items) was used.

Section 4: Italian version of the scale of Integration/Opposition to urban green (Carrus et al., 2004), consisting of two separate dimensions of positive and negative attitudes toward urban green (10 5-step Likert scale items).

Section 5: Socio-demographic data and, for on-site respondents only, an open-ended question asking the amount of hours and/or minutes spent in the environment.

In all sections Likert-type items scored from 1 (“I completely disagree”) to 5 (“I completely agree”).

#### Analyses

Reliability Analyses (Cronbach’s Alpha) were preliminarily carried out, in order to check for the internal consistency of the measures. Then mean scores were calculated for each of the measures used in subsequent analyses. Given the quasi-experimental design of the study, one-way ANOVAs were performed in order to check for any potential difference in personal and experiential characteristics of participants across the three experimental conditions.

The hypotheses testing was made through different analyses:

For H1 and H2, a 3 × 2 repeated measure ANOVA (general linear model), considering the Environment (natural vs. built/historical) as a within-subjects factor, and the experimental Condition (natural vs. built/historical vs. neutral setting) as a between-subjects factor, and the mean score of PRP as the dependent variable;

For H3, a mean score of each restorative property of Villa Celimontana was preliminarily calculated for respondents in the Built/historical and in the Neutral conditions (collectively labeled Not Natural Condition, NNC), in order to have a baseline for a comparison of each component with respondents in the Natural Condition (NC). A difference score from that mean was then calculated for each component and each subject in the three Conditions. One-way ANOVAs contrasting subjects in NC and in NNC were performed in order to evaluate the added value of on-site experience in terms of restorative properties of the Natural Environment. Similarly, a mean score of each restorative property of Piazza Navona was preliminarily calculated for respondents in the Natural and in the Neutral conditions (collectively labeled Not Built/historical Condition, NBC), in order to have a baseline for a comparison of each component with respondents in the Built/historical Condition (BC). A difference score from that mean was then calculated for each component and each subject in the three Conditions. One-way ANOVAs contrasting subjects in BC and NBC were performed in order to evaluate the added value of on-site experience in terms of restorative properties of the Built/historical Environment;

For H4, Hierarchical Multiple Regression Analyses (HMRAs) and Mediation Analyses (MAs) were performed to better understand the relationships between time spent in restorative environments, perception of the restorative properties, and PRP.

#### Results

##### Reliability analyses and self-selection checks

On the whole, the scales employed showed an adequate internal consistency, ranging from 0.67 to 0.81 (see Table 2).

**Table 2:** Reliability analyses (Cronbach’s alpha).

Scale	α
Being away – Villa Celimontana	0.72
Fascination – Villa Celimontana	0.79
Extent – Villa Celimontana	0.67
Compatibility –Villa Celimontana	0.73
Being away – Piazza Navona	0.77
Fascination – Piazza Navona	0.72
Extent – Piazza Navona	0.68
Compatibility – Piazza Navona	0.69
Openness to culture	0.75
Openness to experience	0.69
Experience seeking	0.70
Attitudes toward urban green – Integration	0.81
Attitudes toward urban green – Opposition	0.68

Both the natural (*M* = 3.34, *SD* = 0.76) and the built/historical environment (*M* = 3.23, *SD* = 0.80) scored high in terms of expressed preference by respondents, with no significant difference between them [*F*_(1,124)_ = 1.72, n.s.]. The one-way ANOVAs to check for potential group differences across the experimental conditions were performed for age and the following psychological variables: familiarity with and perceived crowding of the environments; the two sub-scales of the BFQ, namely Openness to Culture and Openness to Experience; the Sensation Seeking sub-scale, namely Experience Seeking; the two sub-scales of attitude toward urban green, namely Integration and Opposition. On the whole, results showed no significant difference between groups, with two exceptions. The first refers to age [*F*_(2,122)_ = 11.55, *p* = 0.000]; the Duncan test for *post hoc* comparisons (for *p* < 0.05) showed that respondents at Piazza Navona were significantly older than respondents in the other two conditions. The second refers to the variable Experience Seeking [*F*_(2,110)_ = 7.26, *p* < 0.001]; the Duncan test for *post hoc* comparisons (for *p* < 0.05) showed that the level of Experience Seeking was higher for respondents recruited in Villa Celimontana. To rule out the possibility of an influence of these variables in the analyses, a check for its relationship with the dependent variables was performed. No significant relationship emerged between age and PRP in Villa Celimontana (*r* = −0.11, n.s.) and Piazza Navona (*r* = 0.06, n.s.). Similarly, no significant relationship emerged between Experience Seeking and PRP in Villa Celimontana (*r* = −0.04, n.s.) and Piazza Navona (*r* = −0.16, n.s.).

##### Restorative potential of natural and built/historical environments and effects of on-site experience on perception of restorative properties and PRP

**FIGURE 2:**
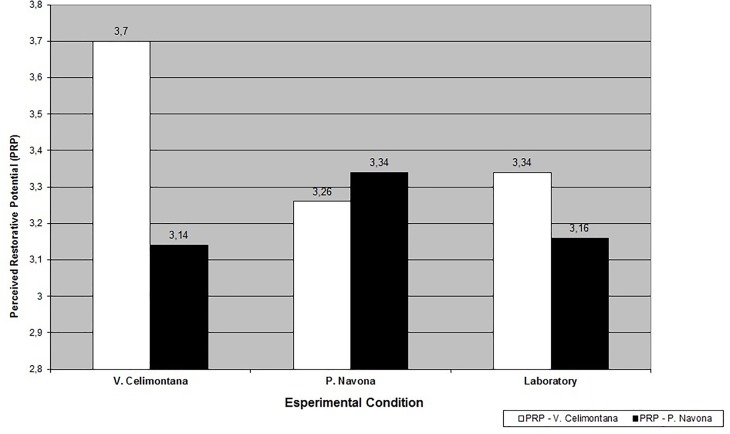
Perceived restorative potential (PRP) of Villa Celimontana and Piazza Navona by experimental condition.

The repeated measures ANOVA showed findings supporting the study’s hypotheses. On the whole, a significant main effect of Environment on PRP emerged [*F*_(1,122)_ = 6.57, *p* = 0.012, η^2^ = 0.05], in line with H1. Villa Celimontana was perceived as more restorative (*M* = 3.42, SD = 0.71) than Piazza Navona (*M* = 3.21, *SD* = 0.68). A significant interaction between Environment and Condition was also found [*F*_(2,122)_ = 4.28, *p* = 0.016, η^2^ = 0.07], in line with H2 (see Figure 2).

In particular, the Duncan test for *post hoc* comparisons (for *p* < 0.05) showed a higher level of PRP of Villa Celimontana among on-site participants compared to the two other conditions. Conversely, no significant difference in the level of PRP of Piazza Navona emerged across conditions. In addition, a higher level of PRP of the natural vs. the built/historical environment [*F*_(1,36)_ = 13.81, *p* = 0.001, η^2^ = 0.28] emerged among participants in Villa Celimontana. For participants in the Neutral condition, the level of PRP of Villa Celimontana was lower if compared to the on-site experience, and no more significantly higher than for Piazza Navona [*F*_(1,49)_ = 1.25, n.s.]. Similarly, for participants in Piazza Navona, no significant difference emerged in PRP of the two settings [*F*_(1,37)_ = 0.47, n.s.].

One-way ANOVAs carried out to explore the effects of on-site experiences on the perception of the restorative properties of environments showed a different pattern of results for Villa Celimontana and Piazza Navona. With reference to Villa Celimontana, the perception of being-away [*F*_(1,123)_ = 5.01, *p* = 0.027, η^2^ = 0.04] and compatibility [*F*_(1,123)_ = 8.96, *p* = 0.003, η^2^ = 0.07] was higher for subjects in NC than in NNC, and the perception of extent showed a tendency to significance in the same direction [*F*_(1,123)_ = 2.79, *p* = 0.098, η^2^ = 0.02]; no significant difference emerged between subjects in NC and NNC in the perception of fascination [*F*_(1,123)_ = 1.33, n.s.] (see Table 3).

**Table 3:** Perceived restorative properties of Villa Celimontana: comparison between NC and NNC.

Component	Environment	*N*	Mean	*SD*	*F*_(1,123)_	*p*
Being-away	NC	37	0.31	0.65	5.01	0.027
	NNC	88	0.01	0.68		
Fascination	NC	37	0.12	0.50	1.33	0.252
	NNC	88	−0.03	0.72		
Compatibility	NC	37	0.40	0.66	8.96	0.003
	NNC	88	0.01	0.68		
Extent	NC	37	0.18	0.39	2.79	0.098
	NNC	88	−0.01	0.57		

*NC, natural condition. NNC, not natural condition.*

With reference to Piazza Navona, no significant difference emerged between subjects in BC and NBC in the perception of the four restorative properties of the environment (see Table 4).

**Table 4:** Perceived restorative properties of Piazza Navona: Comparison between BC and NBC.

Component	Environment	*N*	Mean	*SD*	*F*_(1,123)_	*p*
Being-away	BC	38	−0.01	0.70	0.00	0.988
	NBC	87	−0.01	0.75		
Fascination	BC	38	−0.02	0.58	0.00	0.989
	NBC	87	−0.03	0.59		
Compatibility	BC	38	0.23	0.71	0.07	0.791
	NBC	87	0.20	0.49		
Extent	BC	38	0.09	0.46	1.56	0.214
	NBC	87	−0.02	0.50		

*BC, built/historical condition. NBC, not built/historical condition.*

These results confirmed H3 only for the natural environment, and with reference to the properties of being-away and compatibility.

##### Effects of duration of the experience on the restoration process in natural and built/historical environments

Hierarchical Multiple Regression Analyses and MAs performed to understand the relationships between the duration of the experience in restorative environments, perception of the restorative components, and PRP, yielded different results for Villa Celimontana and Piazza Navona.

With reference to Villa Celimontana, at steps 1 and 2 of the HMRA the duration of the experience and the perceptions of the restorative properties, respectively, were entered as predictors of PRP (Table 5).

**Table 5:** HMRA for Villa Celimontana: effects of duration of the experience and restorative properties on perceived restorative potential (PRP).

Predictors of PRP	β coefficients		Adjusted *R*^2^	*R*^2^ change
	Step 1	Step 2		
*Step 1*			0.17^∗∗∗^	
Duration of experience	0.42^∗∗∗^	0.12^∗^		
*Step 2*			0.71^∗∗∗^	0.54^∗∗∗^
Being-away		0.16^∗^		
Fascination		0.31^∗∗∗^		
Compatibility		0.32^∗∗∗^		
Extent		0.17^∗∗^		

*^∗∗∗^p < 0.001, ^∗∗^p < 0.01, ^∗^p < 0.05.*

At step 1 the model was significant and the duration of the experience emerged as a significant predictor of PRP (β = 0.42, *p* = 0.000). At step 2, the model significantly increased the amount of explained variance, with Being-away (β = 0.16, *p* = 0.014), Fascination (β = 0.31, *p* = 0.000), Compatibility (β = 0.32, *p* = 0.000), and Extent (β = 0.17, *p* = 0.010) as significant predictors of PRP. The duration of the experience was still a significant predictor of PRP (β = 0.12, *p* = 0.034), but its effect on the dependent variable was lower. MAs performed through the Sobel test showed that Being-away (*z*-value = 2.10, *p* = 0.036), Fascination (*z*-value = 2.18, *p* = 0.013), and Compatibility (*z*-value = 3.29, *p* = 0.001) partially mediate the effect of the duration of the experience on PRP. HMRA conducted only on visitors of Villa Celimontana yielded similar results.

With reference to Piazza Navona, at steps 1 and 2 of the HMRA the duration of the experience and the perceptions of the restorative properties, respectively, were entered as predictors of PRP (Table 6).

**Table 6:** HMRA for Piazza Navona: Effects of duration of the experience and restorative properties on perceived restorative potential (PRP).

Predictors of PRP	β coefficients		Adjusted *R*^2^	*R*^2^ change
	Step 1	Step 2		
**Step 1**			0.001	
Duration of experience	0.04	0.09		
**Step 2**			0.27^∗∗∗^	0.27^∗∗∗^
Being-away		0.06		
Fascination		0.31^∗∗^		
Compatibility		0.22^∗^		
Extent		0.20^		

*^∗∗∗^p < 0.001, ^∗∗^p < 0.01, ^∗^p < 0.05; ˆp < 0.08.*

At step 1 the model was not significant. At step 2, the model was significant, and both Fascination (β = 0.20, *p* = 0.035) and Compatibility (β = 0.22, *p* = 0.026) emerged as significant predictors of PRP. The association between Extent and PRP showed a tendency to significance (β = 0.20, *p* = 0.059), while Being-away did not emerge as a significant predictor of PRP (β = 0.05, n.s.). HMRA conducted only on visitors of Piazza Navona showed similar results with reference to the role of restorative properties. In addition, a negative association between the duration of the experience and PRP (β = −0.40, *p* = 0.012) emerged at Step 1.

Taken together, these results confirmed H4 only for the natural environment.

## Discussion

The study yielded preliminary results which can help shed light on some neglected issues in the study of restorative environments. In addition, it can contribute to gain a better understanding of the restoration processes occurring in natural and built/historical environments.

To begin with, a general result emerging from our analysis showed that natural environments can promote higher restoration also when compared to built/historical ones with similar restorative potential. This is the first empirical evidence, to our knowledge, that nature in itself – and not merely because of its restorative properties – is restorative, in line with ART (Kaplan and Kaplan, 1989; Kaplan, 1995), SRT (Ulrich, 1983), and overall the Biophilia hypothesis (Kellert and Wilson, 1993). Past research has widely investigated the beneficial effects of contact with nature (see Bratman et al., 2012; Hartig et al., 2014 for systematic reviews), but it was impossible to disentangle whether these effect were promoted by nature in itself or the restorative properties of natural environments. Moreover, also when considering both typologies of environment, the literature has missed an adequate addressing of this issue, because of the unfair comparison between natural and built environments (Ulrich, 1979, 1981; Hartig et al., 1991, 2003; Ulrich et al., 1991; Purcell et al., 2001; Herzog et al., 2003; Laumann et al., 2003; Staats et al., 2003; van den Berg et al., 2003; Berto, 2005). The generalization of our finding can be questionable, given the specific places we selected for the study, and further comparisons between different examples from both categories should be performed. Nonetheless, the selection procedure we adopted led us to identify two environments with a comparable – and as high as possible – level of perceived restorativeness and aesthetic evaluation/preference, which is strongly associated to perceived restoration (Purcell et al., 2001; van den Berg et al., 2003). Overall, this result adds to the literature on the restorative potential of nature in terms of internal validity, because of the specific comparison made. These results substantially confirmed our expectations as expressed in H1.

Another interesting result has been outlined with reference to the role of place experiences in moderating the perception of the restorative potential of natural and built/historical environments. The influence of on-site experience has clearly emerged, with substantial differences between the natural and the built/historical environment, and in line with H2. Experiencing the environment implies the involvement of a multiplicity of aspects, as theoretically suggested by Canter (1977) and empirically confirmed in a variety of studies (Vining and Orland, 1989; Hull and Stewart, 1992; Scott and Canter, 1997). The more striking result was that on-site experiences increased the restorative potential of the natural, but not the built/historical environment, in line with evolutionary approaches. In the natural vs. built/historical environment comparison across experimental conditions, other interesting results have been found. The gap in favor of the natural environment in terms of perceived restorative potential, as proposed by the evolutionary framework considered, showed to be at the highest level when people were at Villa Celimontana, thus confirming the importance of the immersion in natural environments for a deeper restoration (Kaplan and Talbot, 1983; Kaplan and Kaplan, 1989; Borrie and Roggenbuck, 2001). This difference completely disappeared when people were in the built/historical environment, where on-site experience slightly increased the perception of restorative qualities referring to Piazza Navona, and dramatically reduced the attribution of restorative qualities to the natural environment. In a neutral condition, the effect of actual experience could not emerge, and the evaluation of both environments settled somewhere in between, again with no significant difference among the two. In this regard, Herzog et al. (2002) clearly highlighted the possibility for people to choose non-natural environments as a setting for restoration, stating that in some contextual situations they “might underappreciate the restorative potential of natural activities” (p. 296). Nonetheless, the restorative potential of pleasant built/historical environments has clearly emerged, confirming previous research (Kaplan et al., 1993; Scopelliti and Giuliani, 2004, 2005; Ouellette et al., 2005; Karmanov and Hamel, 2008; Abdulkarim and Nasar, 2014), and suggesting new directions in the study of restorative environments. This study can represent a relevant starting point in this regard, also in line with the recommendation of UNESCO (2011) on the management of Historical Urban Landscapes (HUL).

Some more reflection on the process leading to restoration in both environments can be developed by a deeper analysis of on-site perceptions of the four restorative properties, and the relationships between the duration of the experience, perceptions, and beneficial outcomes. Respondents in the Natural Condition (NC) showed higher levels of perceived compatibility, being-away, and slightly extent of Villa Celimontana compared to respondents in Not Natural Condition (NNC). We can refer to this as to an Increasing Restoration Effect (IRE) of natural experience. Conversely, respondents in the Built Condition (BC) and Not Built Condition (NBC) showed similar levels in the perception of the restorative properties of the Piazza. This result confirmed H3 only for the natural environment, and is still in line with the evolutionary explanations of people-environment transactions. The added value of on-site experience of natural environment in promoting restoration seemed to be strongly grounded on the increase of perceived compatibility, being-away and, to a lesser degree, extent. The final HMRAs and MAs helped gain a better understanding of the restoration process in both environments. For Villa Celimontana, the duration of the experience significantly predicted perceived restorative potential, through the partial mediation of the restorative properties, with the exception of extent. In other words, the longer the experience, the stronger participants get in touch with the natural character of the environment in itself and perceive its restorative properties. Both processes lead to restoration. For Piazza Navona, the restoration process showed to be different. The duration of the experience did not predict perceived restorative potential. For on-site respondents, time was even negatively related to positive outcomes. In addition, being-away was not found to be a predictor of perceived restorative potential. Taken together, these results suggested an inverted U-shaped relationship between time spent and restoration in the built/historical environment. Going further in the experience, the built character of the environment seemed to overcome its restorative properties, and this prevents people from taking psychological distance from everyday routines and obligations, thus buffering restoration. In the built/historical environment restoration basically emerged through the properties of fascination, compatibility, and to a lesser degree extent, with no added value of duration of the experience and no difference across conditions. Again, these results confirmed H4 only for the natural environment, and showed to be compatible with the evolutionary explanations of people-environment transactions. Although Kaplan and Kaplan (1989) clearly claimed that the restoration process may occur through several – and deeper – stages, which undoubtedly require time, the role of the duration of the experience has not received systematic attention in the literature so far. This study has tried to start filling this gap. Future research should be devoted to understand if a temporal dimension in the perception – and the development of beneficial effects – of the restorative properties can be identified for natural environments. The present study seems to suggest that the perception of the restorative properties in built environments is immediately important.

On the whole, this study provided promising insights on the psychological processes which can promote restoration in natural and built/historical environments, involving the perception of the components proposed by ART (Kaplan and Kaplan, 1989) as key mechanisms. The idea of a different role played by the four ART components in fostering restoration was expressly recognized by Laumann et al. (2001), who referred to two different restoration outcomes, namely relaxation, which is highly predicted by being-away, and a “more cognitive restoration” which is “assumed to be highly correlated to preference” and is better predicted by fascination and compatibility (p. 43). Similarly, Herzog et al. (2003) claimed for a different predictive power of the several components on the overall perception of restorativeness. Also Scopelliti and Giuliani (2004) took into consideration the “relative weight” (p. 435) of the four components in mentioning the reasons why people felt restored in different recreational experiences. The same authors also suggested that “restoration may occur in specific settings through different processes, in which different restorative components play a key role” (Scopelliti and Giuliani, 2005, p.221). More recently, Hartig et al. (2014) have stressed that nature affects health through multiple and synergistic mechanisms. We can argue that all restorative environments presumably promote beneficial outcomes through different processes. Our results showed that being-away is a key component in the restoration process in natural environments alone, which are more likely to allow people to take some distance from ordinary aspects of everyday life. If being-away is an environmental characteristic specifically referred to nature, the association with relaxation outcomes postulated by Laumann et al. (2001) can be more strongly framed within SRT (Ulrich, 1983). Built environments, even when showing a high artistic or historical value, do not seem to give the same opportunity. However, it is worth noting that our results emerged from the analysis of people living in an urban context, for whom “built is daily,” and some differences might emerge considering respondents with different residential experiences (e.g., rural residents). In addition, the measure of being-away in PRS does not include items referring to feeling away in ancient times or in a different world, which are all relevant aspects for historical environments (Kaplan et al., 1993). Conversely, the fascinating character of built/historical environments, the perceived compatibility with one’s inclination and, to a lesser extent, the possibility to wander in a wide and coherent space can represent relevant restoration mechanisms in this environmental typology, confirming previous research (Ouellette et al., 2005; Scopelliti and Giuliani, 2005; Abdulkarim and Nasar, 2014). Nonetheless, it is important to note that our analysis of the psychological processes implied in restoration is based on ART and the restorative properties measured by PRS. That does not exclude the possibility to identify further mechanisms in future research.

Some shortcomings of our study should be acknowledged. First, as stressed above, our findings emerged from the analysis of very specific environments, although the selection procedure we employed led us to identify highly restorative, preferred and, in some way, prototypical examples from both typologies. As a consequence, any generalization should be taken with caution. Nonetheless, these results should be used as a stimulating starting point for further research, which should better address the analysis of specific environmental features that contribute to promoting restoration.

A further limitation is represented by the quasi-experimental design adopted. In spite of our checks, which seemed to rule out the possibility of self-selection or strong psychological differences among participants across the experimental conditions, other individual characteristics which were not controlled for in our study might play an important role in restoration processes. A design with random assignment to experimental conditions would help better clarify this issue. At the same time, it is important to underline our intention to analyze restoration processes in a field study, in which people’s experiences would have left as much natural as possible. In this regard, also a completely within-subjects design, with the same respondents evaluating the same restorative environments across conditions, would help better understand the added value of on-site experiences in the restoration process. Moreover, the role of some psychological characteristic of participants in the restoration process may have not adequately emerged because of the small sample size, and deserves further investigation.

Finally, the present research provides some indications for the domain of sustainable urban planning and management, which undoubtedly has connections with human health. A more widespread diffusion of green areas within urban contexts seems to be the more effective path toward psychological restoration. However, under some circumstances, physical constraints (e.g., extreme building density) could make it difficult to pursue such a strategy. As an alternative option, planners and decision makers may want to promote psychological restoration among urban dwellers by increasing the architectural quality of built up public space, such as squares and building facades. Such a strategy might be effective through the enhancement of the restorative properties of urban environments, and the role of fascination, according to our findings, can be highly relevant in the restoration processes in built/historical settings. In this perspective, fascination can be promoted both by changing existing features of public space in a more artistic direction, and through a proper maintenance of high-value artistic/historical urban areas. In addition, the idea of including artistic elements such as statues and sculptures in urban green areas could be considered. This can be particularly important in – but not limited to – cities like Rome, and should not only refer to city centers, but conceived within an integrative approach to biophilic urban design (Kellert et al., 2008). In this regard, future research should try to investigate also the restorative potential of mixed natural/artistic settings. Last, it is important to note that we investigated the role of artistic/historical environments in the restoration process, but the concept of art is wide. Some scholars have recently suggested that playful public spaces can promote positive environmental experiences (Stevens, 2007). Future research should adequately consider the different nuances of art, and their potential role in psychological restoration.

## Ethics Statement

All subjects gave written informed consent in accordance with the Declaration of Helsinki. Our Institution did not require an ethics approval for research, because the Ethical Committee at LUMSA University has been established after our data collection. In addition, according to current guidelines of the Ethical Committee of LUMSA and national guidelines of the Italian Psychological Association (AIP), this study does not require ethical approval. It did not involve any vulnerable group; it focused on respondents’ evaluations of environments through non-invasive methods; data are completely anonymous and it is not possible to identify participants from any resulting report; the use of data (and the data collection in itself) could not cause any damage or distress to subjects involved in the study.

## Author Contributions

MS, GC,and MB developed the research rationale. MS and GC wrote the Introduction and Discussion sections. MS performed the statistical analyses and wrote the Materials and Methods and Results sections.

## Conflict of Interest Statement

The authors declare that the research was conducted in the absence of any commercial or financial relationships that could be construed as a potential conflict of interest.
